# Parent-Adolescent Sexual and Reproductive Health Communication Is Very Limited and Associated with Adolescent Poor Behavioral Beliefs and Subjective Norms: Evidence from a Community Based Cross-Sectional Study in Eastern Ethiopia

**DOI:** 10.1371/journal.pone.0129941

**Published:** 2015-07-13

**Authors:** Yadeta Dessie, Yemane Berhane, Alemayehu Worku

**Affiliations:** 1 Haramaya University, College of Health and Medical Sciences, Harar, Ethiopia; 2 Addis Continental Institute of Public Health, Addis Ababa, Ethiopia; 3 Addis Ababa University, School of Public Health, Addis Ababa, Ethiopia; Merced, UNITED STATES

## Abstract

**Introduction:**

While parent-adolescent sexual and reproductive health (SRH) communication is one potential source of SRH information for adolescents, it appears to be inadequately practiced in Ethiopia. This study was designed to investigate the factors that limit or improve parent-adolescent SRH communication in Harar, Eastern Ethiopia.

**Methods:**

A community based cross-sectional study was done on 4,559 adolescents of age 13–18. SRH communication was measured using a nine-item scale whose response ranged from “not at all” to “always.” Summated composite score ranging from 0–36 was generated; higher score indicates high SRH communication. A median value of the composite score was 4 out of the possible 36 with an Interquartile Range (IQR) of 7. Respondents were ranked as very poor, poor and satisfactory communicators based on 33rd and 67th percentiles values. Generalized ordered logit model was applied to investigate the factors associated with SRH communication.

**Results:**

Results showed that the adolescents who were more likely to practice poor-very poor/very poor SRH communication were those who had poor behavioral beliefs on and poor subjective norms of communicating sexual issues with parents and those who perceived their parents’ reproductive health (RH) knowledge as poor. Nonetheless, the probability of poor-very poor/very poor SRH communication was less with high adolescent-parent communication quality, television co-viewing and discussions, and self-disclosure.

**Conclusions:**

Curtailing the adolescents’ underlying poor beliefs and norms, and improving adolescent-parent communication quality, self-disclosure, and television co-viewing and discussions are essential to engage the parents in sexual and reproductive health education of the adolescents.

## Introduction

Adolescence is a period of transition from childhood to adulthood. It is when several physiological, social, and psychological growth and development are undergoing. Sexual and reproductive growth and development is one of the remarkable changes during this period [1,2], and it is when parents are expected to socialize their children, in which parent-adolescent sexual and reproductive health (SRH) communication is one. The communication helps to transmit values, beliefs and expectations about SRH matters to their adolescents [3–5]. Evidence has shown that the communication protects the young from engaging in risky sexual practices and associated adverse health consequences [6,7]

Africa accounted about four-fifth of the estimated five million young people living with HIV [8], and unsafe abortion due to unwanted pregnancy have been inflicting about one-fourth of the four million unsafe abortion among the adolescents [9,10]. The common reason for acquiring these health problems is lack of basic knowledge on reproductive biology and prevention methods. The prevailing potential sources of SRH information for the young people are their peers whom their knowledge are infirmed/equally ignorant or from school which is blamed for the lack of sustainable behavioral changes or from media and religious institutions that occur infrequently [11–13]. Research showed that the conservative norm and taboos on sexuality, and ill-preparation have largely limited the parents’ involvement on SRH communication with their children. However, remarkably, because of the devastating HIV/AIDS problems, parental engagement has recently been receiving more attention [4,8,11,14].

A review on the magnitude of parent-adolescent HIV/AIDS communication in sub-Saharan Africa reported 8% -80% [4]. Adolescents who communicate SRH matters with their parents less likely to engage in unsafe sex [15]. The communication is associated with adolescent’s age, parental education, and parent types [4,16]. Abstinence, pregnancy and HIV/AIDS are more communicated while condom and other contraceptives are rarely communicated [17].

About two-third of the Ethiopia’s population are young and they are the ones whose reproductive health service health services utilization is low and are the perpetrated with various sexual and reproductive health problems [18,19]. The 2011 Ethiopia Demographic and Health Survey (EDHS) report indicated that 0.2% of females and 0.1% of males within the age 15–19 year were infected with HIV and Sexually transmitted infections(STIs) among females was 1% and 0.2% among males [20]. Further, about one–third of pregnancies occurring during this age are unintended [21].

The government of Ethiopia has set short and long term objectives with strategies that encourage parents’ participation [19,22]. However, little is known about the parental engagement, which would probably be associated with the lack of adequate data. A few existing studies that tried to examine SRH communication have revealed that the communication practice in the range of 20%- 43%, and the factors associated with the communication are parental education, adolescent’s age and living arrangements, type of parents, and parents’ sexual and reproductive health knowledge and attitude [23–29]. Study have showed that SRH communication associated with increased condom used during the first sex about two times [29] and contraceptive awareness by about four times [30].

Thus far, in many of the exiting studies, the studies have examined the SRH communication with *yes or no* responses, which tell the presence or absence of communication than how frequent the communication occurs [24, 25, 26, 27]. In addition, the factors that have been examined so far largely cover the socio-demographic characteristics; they have hardly considered the communication quality, interaction, and other related factors. The objective of this study was to investigate the factors that limit or improve parent-adolescent sexual and reproductive health communication among adolescents in Harar, Eastern Ethiopia. The main hypothesis was that besides demographic factors, parent-adolescent relationship and interaction, and perceptions on communicating about sexual issues with their parents in the perspective of the adolescents’ behavioral beliefs, subjective norms and their perception of comforts to communicate on sexual issues and their parental reproductive health knowledge are important factors in the SRH communication. Further, the adolescent-parent television co-viewing and discussions which have a mediational influence in the young people sexual knowledge and attitude and behavior [31,32] was examined as a factor in this study.

## Methods and Procedures

### Study setting, design and samples

This study was conducted in Harar town found to the east which is 510 km away from Addis Ababa—capital city of the Ethiopia. It has nineteen administrative kebeles. The total population of the town is estimated to be about hundred thousand. Three major ethnic groups living in the town are Oromo, Amhara, and Harari [33,34]. The study used a cross-sectional data of a community based longitudinal study to examine the parenting practices influences on adolescent sexual behaviors. The study was conducted in all the nineteen kebeles. The study has had two waves conducted at about a one year interval. The baseline (Wave-I) survey was conducted in 2012 from March to July and the second round (Wave-II) was conducted after about one year in 2013 from March to mid-August. Respondents were adolescents of age 13–18 year-old primarily living with their parents within the last twelve months prior to the study. Parents in this study denote biological mother/father or female/ male guardians with whom the adolescents were primarily living the last twelve months prior to the study. Every household with the eligible adolescent(s) were considered. When there was two or more adolescents living in a household, one of them was chosen through a lottery method. The baseline survey (Wave-I) addressed 4559 adolescents and all the samples were included in the study. The adequacy of the sample size to meet the objective of this study was checked.

The mean (±SD) age of respondents was 15.4(±1.67) years. More than half 2591(56.83%) were females. The vast majority, 4195 (92.00%) were in-school adolescents. Overall, 1975 (43.32%) of the respondents were secondary and more. Nearly half of the respondents; 2239 (49.11%) were living with both biological father and mother. More than half; 2506(54.97%) were Orthodox Christian in their religion Table 1.

**Table 1:** Brief characteristics of the respondents (N = 4559), Harar, Ethiopia.

Characteristics of the respondents	Categories	n(%)
Sex of the adolescent	Female	2591(56.83)
	Male	1968 (43.17)
Adolescent age in years	13–14 year	1601 (35.11)
	15–16 year	1520 (33.34)
	17–18 year	1438 (31.45)
Adolescent religion affiliation	Orthodox Christian	2506 (54.96)
	Muslim	1609 (35.29)
	Others*	444 (9.73)
Schooling of the adolescents	In-school	4195 (92.01)
	Out-of-school	364 (7.09)
Adolescent living arrangements	With biological mother and father	2239 (49.11)
	With biological mother alone	992 (21.75)
	With biological father alone	155(3.39)
	With female guardian alone	488 (10.70)
	With male guardian alone	117 (2.56)
	With male and female guardian	546 (11.97)
	With others(+)	22 (0.4)

*Catholic, Wakefata and did not have / did not report

^+^biological mother/father living with other persons/individuals

### Data collection

The data were collected using a structured questionnaire prepared in English-language and translated to Amharic and Afan Oromo-languages. The tool was pre-tested among 270 adolescents in Diredawa town which is one of the nearby towns to Harar. Pilot test was done among 60 adolescents in Harar town. Those respondents used for the pilot testing were not considered for the main study. Interviewer administered data collection technique was used. The interviews were conducted at respondents home in the community. The data collectors were thoroughly trained on the data collection procedure, and how to correctly deal with the respondents. Male interviewers interviewed the male respondents and the female interviewers interviewed the female respondents. Thoroughly trained supervisors and the principal investigator supervised the data collection process.

### Measurements

#### Parent-adolescent sexual and reproductive health communication (dependent variable)

This was assessed using a Likert scale of nine items/topics with responses ranging from not at all (recorded as1) to always (recorded as 5) which was derived from the literatures [5,35]. These topics were reported for the respective mother and father and had good internal consistencies (more than 0.81). A sample item includes ‘how often you and your mother/father ever talked about HIV/ AIDS’. All the topics with their frequency distributions are presented in Table 2. Summated composite score was produced through taking the higher from either of the parents where the score was ranging from 9–45 and centered to 0–36 during analysis (by subtracting nine). The median value with Interquartile Range (IQR) was calculated. We then ranked them into very poor, poor and satisfactory communicators based on 33^rd^ and 67^th^ percentiles values.

**Table 2:** Adolescents sexual and reproductive health communications with mother and father, Harar, Ethiopia.

Parent-adolescent SRH communications	Parent type	Frequency of communications				
		Never n(%)	Rarely n (%)	S/times*n (%)	M/times^$^n(%)	Always,n(%)
Communication about choosing boy/ girl friend	Mother(4287)	3208 (74.83)	375 (8.75)	464 (10.82)	187 (4.36)	53 (1.24)
	Father(3079)	2583 (83.89)	229 (7.44)	184(5.98)	73 (2.37)	10 (0.32)
Communication about birth control	Mother(4287)	3550 (82.81)	311 (7.25)	336 (7.84)	80 (1.87)	10 (0.23)
	Father(3079)	2850 (92.56)	134 (4.35)	71 (2.31)	20 (0.65)	4 (0.13)
Communication about condoms	Mother(4287)	3757 (87.64)	270 (6.30)	184 (4.29)	61 (1.42)	15 (0.35)
	Father(3079)	2843 (92.34)	129 (4.19)	79 (2.57)	23 (0.75)	5 (0.16)
Communication about HIV/AIDS	Mother(4287)	1464 (34.15)	97(13.93)	1488(34.71)	590 (13.76)	48(3.45)
	Father(3079)	1433 (46.54)	453(14.71)	829(26.92)	277 (9.00)	87 (2.83)
Communication about reproduction/pregnancy	Mother(4287)	3167 (73.87)	566(13.20)	399 (9.31)	132 (3.08)	23 (0.54)
	Father(3079)	2645(85.90)	240 (7.79)	142 (4.61)	45 (1.46)	7 (0.23)
Communication about reproductive organ growth and development	Mother(4287)	2428 (56.64)	715 (16.68)	780 (18.19)	326 (7.60)	38 (0.89)
	Father(3079)	2230 (72.43)	394 (12.80)	359 (11.66)	84 (2.73)	12 (0.39)
Communication about Sexually Transmitted Infections^+^	Mother(4287)	3275 (76.39)	488(11.38)	400 (9.33)	10 (2.47)	18(0.42)
	Father(3079)	2574 (83.60)	249 (8.09)	192(6.24)	56 (1.82)	8(0.26)
Communication about how to handle sexual pressure from friends or potential partners	Mother(4287)	3077 (71.78)	367(8.56)	450 (10.50)	318 (7.42)	7(1.75)
	Father(3079)	2535 (82.33)	209 (6.79)	195 (6.33)	117 (3.80)	23(0.75)
Communication about when to start having sex	Mother(4287)	3343(77.98)	333 (7.77)	318 (7.42)	221(5.16)	72(1.68)
	Father(3079)	2633 (85.51)	183 (5.94)	152 (4.94)	82 (2.66)	29(0.94)

*Some times,

^$^ most of the time

^+^ other than HIV/AIDS

#### Adolescent self-disclosure to parents

We examined this using a Likert of five items that measured the extent of adolescent self-disclosure of activities to their parents with responses that ranged from strongly agree (1) to strongly disagree (5) derived from the literature[36]. The scale had internal consistency of 0.78. A sample item includes ‘you are keeping secrets from your mother/father about what you do during your free time’ (reversed during analysis). A summated composite score was generated where higher indicates greater level of self-disclosure and was classified into low or high using mean.

#### Adolescent-parent general communications quality

We examined this using two items on the extent to which adolescents feel easy to communicate about any problems/ issues they face in their daily encounters with the mother and father derived from the literature [32]. A sample item includes ‘you find easy to discuss any problem with your mother/father’. The responses were ranging from strongly disagree (1) to strongly agree (5). The scale has internal consistencies of more than 0.61. We generated a summated composite score by taking the higher of the communication from either of the parents where higher indicates greater communication quality and then classified into low or high using mean value.

#### Adolescent-parent-television co-viewing and content discussions

We assessed these by a five item Likert scale with the responses that ranged from strongly disagree agree (1) to agree represented (5) derived from the past literature [32]. A sample from the items included ‘your mother/father talk with you to help you understand what you have watched on television’. The internal consistency of the scale was 0.70. We generated a summed composite score and then categorized into low or high based on the mean, and those did not have television were coded as did not have television.

#### Adolescent behavioral beliefs to communication on sexual issues with the parents

We applied four item Likert scale to examine adolescents’ behavioral beliefs about possible consequences to communicate about sexual issues with the parents [37]. The items have responses ranging from strongly disagree agree (1) to strongly agree (5). A sample item was ‘you think your mother/father is afraid of encouraging sexual activity if they talk to you about sexuality’. The scale had internal consistency of 0.65. We have generated a summated composite score that the higher indicate lesser behavioral beliefs. It was then classified into good and poor beliefs using mean value.

#### The adolescent perceived subjective norms

We assessed the subjective norms using two item Likert scale which asked the adolescent perception on how other people important to them(close friends) think he/she has to communicate on sexual issues with parents derived from the literature [37]. The items responses were ranging from strongly disagree (5) and strongly agree (1). A sample from the items was ‘your close friend thinks you should talk about sexuality with your parent’. The scale internal consistency was 0.63. We generated a summated composite score where the higher indicate poorer subjective norm, and we then classified it into good or poor subjective norms using the mean value.

Other covariates were the level of comfort to communicate on sexual issues with the parent as classified as comfortable and uncomfortable; adolescent perception on parental reproductive health knowledge classified into know very well, know to some extent or poor; age of the adolescents classified into 13–14 year, 15–16 year or 17–18 year; sex of the adolescents as male or female; schooling classified into in-school or out-of-school; adolescent religious affiliations classified as Orthodox Christian, Muslim or others; adolescent living arrangements classified into living with both the mother and father, mother alone, father alone, female guardian alone, male guardian alone, and male and female guardian or others; adolescents and parental educational levels were classified into primary and less, and secondary and more.

### Statistical analysis

The data were double entered, validated and cleaned using EpiData software Version 3.1. The data were analyzed using STATA Version 12(College Station, TX: StataCorp LP). Generalized ordered logit model (gologit2) was applied for fitting the model due to the parallel assumptions of the ordinal logistic regression (ologit) fail to meet. Generalized ordered logit model be able fits models that are less restrictive than (ologit) coefficients [38]. The equations are presented below to make the explanation plain.

$$\text{P}(\text{Yi }>j)=\frac{\text{exp}\left(\alpha \text{jX}1i\beta 1+\text{ X}2\text{i}\beta 2+\;\text{X}3\text{i}\beta 3\text{j}\right)\;}{\left\{\text{exp}\left(\alpha \;+\text{ X}1\;\beta 1+\text{ X}2\;\beta 2+\text{ X}3\;\beta 3\;\right)\right\}},\text{j }=1,\;2\ldots \text{ M }–\;1$$ (1)

In Eq 1 above, the beta (β) coefficient indicates association of the independent variable for which some ‘betas’ can differ across levels of ‘ j’ but others do not. This means that β1and β2 are the same and β3 can vary. In the equation alpha (αj) represents a cut off point for the j^th^ cumulative logit.

$$\text{P}(\text{Yi }>j)=\frac{\text{exp}(\alpha \text{j}-\left[\text{ X}\beta \text{j}-\text{T}\gamma 3\text{j}\right)}{\left\{1-\text{exp}\left(\alpha \text{j}-(\left[\text{ X}\beta \text{j}-\text{T}\gamma 3\text{j}\right)\;\right)\right\}},\text{j }=1,\;2\ldots \text{ M }–\;1$$ (2)

In Eq 2 above, the gamma (γ) parameterization indicates the differences in proportionality of the’ β-coefficient’ at different ‘j’ levels that can be either significant or insignificant/not appear. When the gamma did not appear or insignificant, it indicates the association factor variable (β-coefficient) remain the same across ‘j’ levels. Whereas, when gamma (γ) found to be significant, it indicates the β- coefficient is varying across ‘j’ levels. In this case, it means that the rank from ‘satisfactory’ to combined poor and very poor represented as’ poor-very poor’ or from combined ‘satisfactory and poor’ to very poor communication level. In this case, the β-coefficients of a variable which explains very poor versus the ‘satisfactory and poor communication’ was obtained by adding the ‘β’ and ‘γ’ coefficients.

The bivariate generalized ordered regression was fitted and variables found to be significant at p-value <0.05 were entered to multivariable model. In bivariate model, different variables had violated the proportional odds assumptions which have significant p-values in their gamma coefficients Table 3. In the final model Table 4 when adjusted, the variables or variable/s categories that violet meet the parallel assumptions were sex, age, Muslim religion, living with mother alone, high self-disclosure to parents, adolescents’ poor beliefs and subjective norms. In these cases, the respective variables associations were obtained by adding ‘β’ and ‘γ’ coefficients which indicate the association at Eq 2 (with very poor communication) versus combined ‘satisfactory and poor communication’. Statistical significance was declared at (p <0.05).

**Table 3:** Bivariate result of factors associated with parent- adolescent sexual and reproductive health communication in Harar, Ethiopia.

Factors	Categories	Parent-adolescent SRH communications				Unadjusted generalized ordered logit model			
		n(%)	Satisfactory (%)	Poor (%)	Very poor (%)	Beta (β)	P-value	Gamma-2(γ)	P-value
Sex of the adolescents	Female	2591 (56.83)	39.98	32.88	27.13	1		1	
	Male	1968 (43.17)	18.95	46.49	34.55	1.046	<0.001	-0.697	<0.001
Age in years of the adolescents	13–14 year	1601 (35.11)	24.55	42.54	32.92	0.620	<0.001	0.371	<0.001
	15–16 year	1520 (33.34)	31.18	38.68	30.13	0.288	<0.001	-0.169	0.040
	17–18 year	1438 (31.45)	37.69	34.63	27.68	1			
Adolescent religion affiliation	Orthodox Christian	2506 (54.96)	33.16	41.46	25.38	1		1	
	Muslim	1609 (35.29)	25.17	34.43	40.40	0.392	<0.001	0.292	<0.001
	Others	444 (9.73)	38.96	39.19	21.85	-0.231	0.016		
Schooling of the adolescents	In-school	4195 (92.01)	31.23	39.07	29.70	1			
	Out-of-school	364 (7.09)	27.20	35.16	37.64	0.288	0.005		
Adolescent living arrangements	With biological mother and father	2239 (49.11)	33.50	41.85	24.65	1		1	
	With biological mother alone	992 (21.75)	31.35	34.48	34.17	0.134	0.095	0.278	<0.001
	With biological father alone	155 (3.39)	23.87	38.06	38.06	0.554	<0.001		
	With female guardian alone	488 (10.70)	23.57	35.86	40.57	0.628	<0.001		
	With male guardian alone	117 (2.56)	36.75	32.48	30.77	0.057	0.749		
	With male and female guardian	546 (11.97)	26.74	38.10	35.16	0.414	<0.001		
	With others	22 (0.4)	31.82	36.36	31.82	0.209	0.602		
Educational status of the parent	Primary and less	2493 (54.68)	25.71	37.38	36.90	1		1	
	Secondary and more	2066 (45.32)	37.17	40.42	22.41	-0.536	<0.001	-0.169	0.016
Television co-viewing and discussion	No-television	387 (8.48)	20.67	38.24	41.09	1			
	Low	2263 (49.20)	26.34	38.00	35.66	-0.264	0.010		
	High	1909 (41.87)	38.40	39.76	21.84	-0.879	<0.001		
Adolescent self-disclosure to parent	Low	2051 (44.98)	29.40	35.15	35.45	1		1	
	High	2508 (43.02)	32.14	41.71	26.16	-0.128	0.047	-0.309	<0.001
Perception on parent RH knowledge	Very well	1175 (25.77)	47.44	36.71	15.84	1			
	Some extent	2855 (62.62)	27.76	39.54	32.70	0.887	<0.001		
	Poor	529 (11.60)	12.27	38.64	49.08	1.883	<0.001	-0.299	0.024
Comfort to communicate on SRH issues with the parents	Comfortable	1945 (42.26)	50.85	33.01	16.14	1		1	
	Uncomfortable	2614 (57.33)	16.07	43.04	40.90	1.687	<0.001	-0.407	<0.001
Adolescent-parent-communication quality	Low	2437 (53.45)	17.89	40.87	41.24			1	
	High	2122 (46.54)	45.85	36.33	17.81	-1.357	<0.001	0.182	0.016
Adolescents behavioral beliefs	Good	2049 (44.94)	45.53	32.16	22.30	1		1	
	Poor	2510 (55.06)	18.96	44.14	36.89	1.273	<0.001	0.562	<0.001
Adolescents subjective norms	Good	1949 (42.75)	48.85	30.48	20.68	1		1	
	Poor	2610 (47.25)	17.51	44.94	37.55	1.503	<0.001	-0.668	<0.001

**Table 4:** Multivariable generalized ordered logit model of factors associated with parent-adolescent sexual and reproductive health communication in Harar, Ethiopia, 2012.

Factors	Categories	Adjusted generalized ordered logit model			
		Coefficients	95% CI		P-Value
		Beta (β)			
Sex	Male	0.745	0.586	0.903	<0.001
Age	13–14 year	0.607	0.422	0.793	<0.001
	15–16 year	0.342	0.164	0.519	<0.001
Adolescent religion affiliation	Muslim	0.505	-0.110	0.211	0.538
	Other	0.072	-0.132	0.276	0.491
Schooling	Out-of-school	0.029	-0.195	0.255	0.795
Adolescent living arrangements	Biological mother alone	-0.024	-0.210	0.162	0.801
	Biological father alone	0.321	-0.002	0.645	0.050
	Female guardian alone	0.313	0.104	0.522	0.003
	Male guardian alone	-0.061	-0.439	0.317	0.752
	Male and female guardian	0.362	0.169	0.556	<0.001
	Others	0.621	-0.206	1.447	0.141
Parent educational level	Secondary and more	-0.147	-0.279	-0.014	0.031
TV co-viewing and discussion	Low	0.079	-0.139	0.299	0.476
	High	-0.236	-0.463	-0.011	0.040
Adolescent self-disclosure	High	0.112	-0.041	0.265	0.152
Perception on parents RH knowledge	Some extent	0.689	0.544	0.834	<0.001
	Poor knowledge	1.109	0.885	1.334	<0.001
Comfort to communicate with parent	Uncomfortable	0.836	0.702	0.970	<0.001
Communication quality	High	-0.625	-0.754	-0.496	<0.001
Adolescent behavioral beliefs	Poor	0.394	0.230	0.559	<0.001
Adolescent subjective norms	Poor	0.754	0.588	0.918	<0.001
		Gamma-2(γ)			
Sex	Male	-0.642	-0.818	-0.4651	<0.001
Age	13–14 year	-0.312	-0.514	-0.110	0.002
	15–16 year	-0.221	-0.416	-0.025	0.027
Adolescent religion affiliation	Muslim	0.457	0.292	0.622	<0.001
Adolescent living arrangements	Biological mother alone	0.291	0.105	0.475	0.002
Adolescent self-disclosure	High	-0.295	-0.461	-0.129	<0.001
Adolescents behavioral beliefs	Poor	-0.226	-0.421	-0.056	0.010
Adolescent Subjective norms	Poor	-0.519	-0.702	-0.338	<0.001
		Alpha(α)			
Cons-1		-1.018	-1.346	-0.690	<0.001
Cons-2		-2.290	-2.629	-1.950	<0.001

N = 4559, Log likelihood = -4177.000, Pseudo- R^2^ = 0.161, CI = Confidence Interval

### Ethics Statement

The study was reviewed and approved by the Institutional Research Ethics Review Committee (IRERC) of the College of Health and Medical Sciences, Haramaya University. Written consent from parents and then written assents from adolescents were obtained after explaining the design, the objective and benefit of the study. Respondents were clearly informed that participation is voluntary. It was further emphasized that their responses are confidential, and had their right to withdraw from the study any time without giving further explanation. Privacy and confidentiality were resolutely kept in all data collection procedures.

## Results

From the nine items examined, HIV/AIDS was reported as the more frequent topic of communication both with mothers 2,226 (51.92%) and fathers 1193 (38.74%) at a frequency of sometimes and more. Reproductive organ growth and development was second, communicated by 1144 (26.68%) with mothers, and by 455 (14.77%) with fathers. Condom was the least communicated topic. More communications were made with the mothers compared to the father Table 2. Overall, the median value of the summated SRH communication composite score was 4 out of the possible 36, which corresponds communicating on one topic at a frequency of ‘always’ or on two topics at a frequency of ‘sometimes’, or on two topics one at frequency of ‘most of the time’, and another ‘rarely’. Generally, about one-third (30.91%) of the adolescents were identified as satisfactory communicators, 38.76% as poor communicators, and 30.34% as very poor communicators.

In the bivariate analysis, Table 3, the variables that were positively associated with poor- very poor / very poor SRH communications were adolescent’s sex, age, religion affiliation, living arrangement, schooling status, perception of his or her parents’ RH knowledge, comfort status to communicate on SRH, and underlying beliefs and subjective norms. The factors that were inversely associated with poor-very poor / very poor SRH communication were adolescent-parent general communication quality, television co-viewing and discussions, parental educational status, and adolescent self-disclosure to their parents.

As shown in Table 4, the multivariable model showed that male adolescents experienced more poor- very poor / very poor communication compared to the females (β = 0.745; P-value<0.001). Its association at Eq 2 (the association of being male with very poor communication by taking the higher level as a base) was (β = 0.103; P-value = 0.193). The 13–14 year- of-age adolescents were more likely to experience poor-very poor communication compared to those 17–18 year (β = 0.607; P-value<0.001) and the association of this age category at Eq 2 was (β = 0.295, P-value<0.001). Again, poor-very poor SRH communication was higher among 15–18 year adolescents (β = 0.342; P-value<0.001). At Eq 2, it was (β = 119; P-value = 182). Those who were living with father alone (β = 0.328; P-value = 0.050) and with female guardian alone (β = 0.312; P-value = 0.003) were more likely to experience poor-very poor / very poor communication compared to those living with both biological parents. Further, the probability of very poor communication was high among those respondents living with biological mother alone (β = 0.270; P-value = 0.004).

The result furthermore showed that, the adolescents who perceived their parents had poor SRH knowledge (β = 1.109; P-value<0.001), and those who were uncomfortable to communicate sexual issues (β = 0.836; P-value<0.001), were more likely to experience poor-very poor/very poor communication. The adolescents’ poor beliefs to communicate on SRH issues with their parents more likely associated with poor- very poor communication (β = 0.394; P-value<0.001). The association with very poor communication at Eq 2 was (β = 0.156; P-value = 0.054). Moreover, adolescents’ poor subjective norm more likely associated with very poor and poor communication (β = 0.754; P-value<0.001). The association at Eq 2 was (β = 0.233; P-value = 0.005) (Table 4).

The probability of poor-very poor/very poor SRH communication were less among adolescents who had high quality general communication (β = -0.652; P-value<0.001), and high television co-viewing and discussions (β = -0.237; P-value = 0.041) with their parents. Adolescents’ high self-disclosure to parents was associated with less probability of very poor SRH communication (β = -0.183; P-value<0.014). More results are presented in Table 4.

## Discussion

The topic level descriptive analysis showed that HIV/AIDS was more communicated and that 51.92% of the adolescents communicated with their mothers and 38.74% with their fathers at a frequency of sometime to always. This builds on the report by Tesso and his co-authors [24] from Ethiopia and on other reports elsewhere in Africa [39,40] which have indicated that HIV/AIDS is a common topic. The explanation of this likely linked with the ongoing HIV/AIDS intervention activity, which is under the attentions of many sectors [4]. What could have been important, but not incorporated were the context in which the communications occur and what aspects of the topic have been communicated. Here, linked with the HIV/AIDS communication, we expected condom to be a common communication topics, but we found it was the least communicated topic. In fact, earlier literature showed that most the communications on HIV/AIDS were more focused on the dangerous aspects of the disease than the transmission modes and prevention methods [29].

In this study, the median value of SRH communication was 4, which indicates very limited communication. Previous studies in the country have also indicated an aligned finding that SRH communication is infrequently occurring and often compounded by discomforts [23,24]. The main reasons for the very limited communication could be conservative norms around sexuality, limited parental RH knowledge, and fear that such communication would encourage sexual activities. It is important to note that making comparison is not easy due to inconsistent measurements of parent-adolescent SRH communication in literature [4,5].

We found out that the male adolescents were about 0.75 times more likely to experience poor and very poor communication compared to the females. This is consistent with past literatures that SRH communications occur more among females [17,23,41]. One possible reason is that the females are usually more exposed to SRH adverse outcomes compared to males and this would force the parents to channel more information to their daughters. In addition, the evidence that male adolescents have less motivation to seek such information from their parents than the female might also explain the finding [37].

Like a study finding in west Wollega, Ethiopia [23], in our research, SRH communication was less among the younger age adolescents. This may imply that the parents think their children are liable to risky sexual activities when the children become older. However, because numerous adolescents engage themselves in sexual activity at an early age [42], and this deprives the adolescents chances to get the necessary information on the required time.

In this study, the probability of poor-very poor SRH communication was more likely by about 0.39 times among the adolescents who had poor beliefs, and by about 0.75 times among those who had poor subjective norms. This finding is in due way with the findings of Schouten et al. [37], in which the adolescents’ beliefs and subjective norms were strong predictors. This might go with a theoretical ground that an individual’s attitudes and intentions significantly influence his or her actions [43,44]. Consequently, adolescents may hesitate to seek such information from their parents and the intimate people/friends when environments fail to motivate them. This implicates there is a need to awake the parents’ attitude and reaction of them in such perspective has magnificent implication to make their adolescents seek such information from them. Further, there is a need to aware the adolescents from where to get valuable information on the sexual and reproductive health.

The adolescents who considered their parents’ knowledge of RH as poor were less likely to experience SRH communication with them. This is in concordance with a past research report which confirmed that the level of parental knowledge is largely correlated with the presence of SRH communication [45–47], and with a study finding in Ethiopian, where 21.8% of the adolescents have no interest to communicate with their parents because they consider they have no SRH knowledge and the skill of communication [27]. This calls for an urgent need to equip the parents at least with the basic knowledge and skills of sexual and reproductive health. Here, it is important to note that this study addressed the adolescents’ perceived RH knowledge, which may not reflect the actual RH knowledge of the parents.

In line with the premises that good general communication quality can create easy environments that possibly motivate adolescents experience SRH communication [48]. This study demonstrated that the probability of poor- very poor /very poor communication was less by about 0.62 times among the adolescents who experienced high general communication quality with their parents which were also evidenced elsewhere [4,48].

Literature indicated television co-viewing and discussions among the adolescent and the parent have played mediational influence on young people sexual knowledge and attitude [31]. In this study, it was inversely associated with the probability of poor-very poor /very poor SRH communication. Such practices can produce a platform environment to communicate on sexual issues and what was televised during the co-viewing might also contain topics on sexual issues that can activate such conversation to occur.

In addition to what have already been mentioned, we feel our study has some limitations. One is that it dealt only with the adolescents’ perspective, which might be different from what their parents might really have perceived, so assessing the matter from the perspective of both would provide a broader picture. The other is this study examined only the frequency per certain items, which was very specific and it is important to incorporate the communication contexts and possible contents in each topic to expand to the various domain of the communication. As the adolescents were asked for communication events that might occur some time ago, especially for adolescents of older age, memory bias limitation might occur. Furthermore, associated with the intrinsic challenges of the reporting scale, the respondents might have perceived the distance between the rating values as unequal distant that might have had an effect therefore using a visual analogue scale may better address such issue [49].

## Conclusions

Adolescent-parent SRH communication was largely limited among adolescents who had poor behavioral beliefs, and poor subjective norms to communicate on sexual issues with the parent, and perceived the parents had poor SRH knowledge. Nonetheless, high adolescent-parent general communication quality, television co-viewing and discussion, and adolescent self-disclosure substantially improve the communication. Therefore, to engage the parents in sexual education of the adolescents, improving underlying beliefs and norms, and improve the adolescent-parent communication, self-disclosure and television co-viewing and discussions are essential.
